# Clinical Cases

**Published:** 1895-10

**Authors:** F. O. Pease

**Affiliations:** Chicago, Ill.


### CLINICAL CASES.

F. O. Pease, M. D., Chicago, Ill.

### Case of Mary K.

Age fifteen years, nearly; dark brown hair, gray eyes; in health, a plump and well-developed figure of medium height. Father tall, slender build; slightly stooped; nervous temperament. In general good health, but now sick with typhoid fever—ninth week. Also a nine-year-old sister, convalescing. One sister, thirteen years old, died 9th of October from typhoid fever. Patient's mother in good health usually; of neurotic temperament and given to neuralgias, etc. Patient was always a healthy child, except skin troubles, and grew rapidly last three years; was plump; weighed 145 pounds; healthy and strong up to time of sickness. Menses first in fourteenth year; period due on October 15th: did not appear. October 25th took a severe cold, and complained much of feeling so tired and weak; thought it was due to non-appearance of menses and the cold; tried to use syringe and hot-water douche to bring on flow, but could not insert tube because of painful ulcer (size of a dime), sore and raw-looking on the mucous surface of left labia. This healed in a few days; Carbolic-acid bathing (a white membranous slough came off, leaving raw surface, which slowly healed); about the 31st of October or 1st of November she became delirious at night, and was put to bed on November 2d. On the 5th a crack in skin over tip of coccyx appeared; was very delirious; "wanted to go home;" would jump up and try to get out of bed to go home; must catch a train; called for and talked with the sister who died October 9th, and saw others of her friends; must get them food or a place to sleep, and would seem as exhausted as though the work was really being done. She coughed from the first appearance of the cold.

Nostrils stopped up and hearing disappeared; was very restless; was kept in a stupid quietness by doses of Morphia and Antipyrine. During this time blood had settled around the crack in skin over coccyx, which had turned black, hard, and dry. Wherever the flesh was pressed upon or handled even carefully, ecchymosis began at once, especially about hips, heels, and crest of ilii.

In the course of four or five days the skin got black and hard over coccyx, then dropped out, leaving a gaping ulcer. This was washed out with Carbolic-acid water, and Ammonia water for bathing the body was used, and also Iodoform. These were continued to December 26th. Iodoform was used when or wherever the skin broke open or the black and dried sloughs came away.

Discharges yellow, dark, watery, and offensive. Poultices of Flaxseed meal, compresses of Soda-water, etc., were also used, and Peroxide-hydrogen injected into ulcers. Was vaccinated eighteen months ago; arm ulcerated and was greatly swelled.

Always has had tendency to skin troubles, boils, and pimples. Discharges offensive from bowels or bladder, and menstrual flow offensive, skin dirty; must bathe often to keep body clean. Had in childhood measles, whooping-cough, mumps, and chicken-pox.

The house is situated over an old, poorly-drained slough; lived in since June last by the family.

About a week before the ulcer on labia patient slept with a neighbor girl of fifteen years, who was disliked by mother of patient and not allowed to come again. Patient seems to be and always had been a pure-minded child; had worked during summer in a boarding-house; not away from home nights.

During sickness no appearance of menses, now four months. Under care of three different allopathic physicians, and for just nine days a homœopathic M. D., who continued the Iodoform, poultices, and Peroxide-hydrogen and syringing; *none of the physicians had ever looked at or examined the sores or skin of pa-*

*tient.* All unanimously said that the sores were of slight moment—were only from pressure.

On December 25th the homœopath prognosed probable fatal ending within a day or two.

On December 26th the mother brought to me the above report, which I gathered by careful questioning, and also the following statement of her condition on December 26th. From this point in the case I give report of case and treatment by dates. Patient being in suburbs, reports were daily up to the 28th, when I saw her for the first time.

December 26th, 1893.—Present condition, high fever, 104 F. on eve of the 25th; skin dry, rough, and scaly; tongue dry, shriveled, hard, and dark; thirst marked, large drinks, worse at night, with much restlessness when awake, quiet in sleep; bowels constipated; stools have been light or grayish in color; urine very scanty, dark, strong odor, not frequent, passed unconsciously, has had all along white sediment like thick milk; some milky discharge from vagina; when awake is delirious, at times momentary consciousness and again at times quiet, or is restless with arms, pulls at the bed-covering; motion causes complaint of pain and soreness; breath cadaverous; picks at nose much; swelling of feet and ankles; if turned on left side hands get cold and purplish; the sores *look* worse on the right side but *discharge* more from left side; abdomen sunken, not sore to touch; sores or ulcers over hips, sacrum, coccyx, up the spine, and on left heel. From this description, and after consulting with my colleague, Dr. E. W. Sawyer, I concluded to send Phos-acid $^{2x}$, a drop in a tablespoonful of water once in four hours, before taking her food; also to remove all dressings and poultices from the ulcers, wash clean with soap and water; to put on only dry dressings of absorbent cotton, and to remove all Iodoform, Carbolic-acid, etc., etc., from the room, and to report to me in the morning.

December 27th.—Resting easy this morning; slept from one to two hours during the night. Said she felt sleepy this morning, but has been awake and quiet. Tongue is moist and lighter in color, and the surface of body is more natural; the odor from the sores or discharges very bad, and she seems very weak.

I send Cinchona-off.[1x] gtts. 2 in water every four hours. Report again in the morning.

December 28th.—" Mary was very restless up to one o'clock A. M. Complained of her back. No urine from 9 A. M. to 1.10 last night. Bowels not moved for three days. Seemed to relish the corn-meal gruel; some chicken broth and a glass of egg lemonade yesterday and also this morning. Bubbling noises from the ulcer on right hip last evening for a time. Picks her nose much, and face flushes in the evening. Tongue clearer and moist." This was the morning report. I saw the patient at 6 P. M. for the first time; found her with face flushed, expression somewhat wild, pupils dilated, temperature 100° F., pulse 140, weak but regular; tongue rather clean, moist; breath from nose offensive, not so from mouth; nostrils dry, scabbed, and black inside; emaciation of body and legs marked; skin dry, rough, and scaly, in fine lines; dark ecchymosed broad lines around the limbs and knees, as if skin had parted under the epidermis, sore to touch.

Coccyx naked and exposed for two and one-half inches, in the middle of the cavity, which was three and one-half or four inches long by two broad. Bone looking dry and black except tip, which was dead white. Ulcerous, circular openings over each segment of saccrum at the left border, and increasing in size from below upward, and ulcers up the spine over the spines of vertebræ. Deep opening at border of left crest of ilium one inch deep by one and one-half broad, the cavity black, dry, with grayish spots, oozing pus; on the flank below this, posterior to the left trochanter, four openings to deep broad abscess or pus pockets profusely discharging yellowish creamy pus (this had changed from watery, dark, on the 26th). The tissues felt doughy or boggy for an area of four or five inches, skin bluish or leaden color. The right trochanter was protruding, dry, black, and covered with a thick sphacelous or mass of dead tissue (adherent) from an opening four or five inches long from above downward, and three or three and one-half inches from before backward, pus burrowing among the emaciated muscles, the opening gaping widely.

I proceeded to syringe out all openings and to wash the parts with boiled water, applying dry absorbent cotton dressings, with orders to change as often as necessary, and gave the patient a single dose of Pyrogen$^{cm}$, and stopped the Cinch-off. Placebo once in three hours.

May 9th.—No menses. Felt yesterday as if to be unwell, but no "show." Is gaining flesh and strength rapidly, can almost straighten limbs, is happy and cheerful, cheeks red and rounding out. Cannot or will not bear weight on feet, but continues the use of bicycle. Lac-vac.$^{def.\ cm}$, six powders, one dose at night.

May 21st.—Has become nervous lately, worse in the bad weather (thunder storms), easy tears, won't try her weight on feet. White mucous sediment in urine. Some loose stools, one was greenish. No pain with this diarrhœa. Phos-ac$^{1m}$, one dose.

May 29th.—Is moving to Kempton, Ill. No signs of menses. Cords of legs still shortened; tries to bear weight, but is afraid to; heat in feet, nights; hungry between meals. Sulph.$^{1m}$, four powders, one each morning.

June 12th.—By letter. Menses came on 9th (or 8th) natural. On 10th, after a drive, at midnight was waked with pain in bowels, griping, loose stools; soreness on Monday, 11th; could not eat; feverish and in bed all day. Diarrhœa like pus (?) and offensive. I sent China$^{1m}$, to be taken after stools if not better.

June 16th.—Report: General improvement began spontaneously. She did not get the China. Can walk a little without help, and almost upright.

December 29th.—Report quiet sleep most of the night. Urine scanty, but more frequent and involuntary. The skin is more moist and mind more clear. Pus lighter colored and less offensive. Temperature, 100.7. Pulse, 112. Tongue, red streak in middle and dry tip. ℞. Placebo.

January 1st, 1894.—Marked improvement in general. The coccyx nearly covered with granulation tissue and the flabby gaping is less—*i. e.*, the coccygeal opening is smaller and less patulous.

Other openings, as that over left cresti illii nearly filled with granulations. The large cavity around right trochanter does not sag or gape so widely, and discharges not so profuse from sinuses on left flank and scarcely any odor. Tongue moist and clean, no red streak. Bowels moved spontaneously Saturday night with knowledge of patient, and natural odor and form. Complains of fidgety and restless feet. Is hungry and relishes food. Pulse, 106; temperature, 99. Placebo.

January 3d.—Report of mother. General improvement is steady, and she brings me the "caps" from the spines of the coccyx, two of them that had rested on the top of granulation tissue over coccyx, which is covered. Urine scanty, but twice in the twenty-four hours, still involuntary not unconsciously. Good appetite. Complains much of feet, and wants them moved often. Still lies on back. I send Zinc$^{1m}$, one dose, and Placebo.

January 5th.—Good sleep and appetite. Can lie on left side. Tries to pull off bandage on right hip in sleep. Another piece of bone came off coccyx yesterday, granulations continuing. Memory still clouded, urine unconsciously passed. Strong odor from right hip abscess, large slough separating. Tongue red but moist. Pulse, 112. ℞. Pyrogen$^{cm}$, one dose.

January 9th.—Reported on the 6th. Monday morning: Suppression of urine or at least no urine from Sunday morning. Increase of delirium. I sent Hyos$^{1m}$, a dose in water at 2, 4, and 6 P. M. Passed urine at five o'clock unconsciously. In sleep is restless, when awake complains of a board under her back. (The patient herself clipped off the slough from the right trochanter yesterday.) From the abscess on left flank a slough or mass of tissue (like candle-wicking) was removed also. These sloughs took away with them the offensive odors, leaving now the discharges from all sources almost odorless and creamy. Pulse, 102. ℞. No medicine.

January 12th.—General improvement, urine free and profuse in the aggregate. Saturday up in chair; yesterday, for the first time since sickness, mind clear and natural, except memory only reaches back to recent events. Discharges of pus only from the deep abscess on left flank; she can lie on either side. No medicine since Hyos. of Monday, 10th. As she complains of backache at night and relief from change of position, is not thirsty; the tongue is red at the tip, and weather is damp and stormy, I gave one dose of Rhus.$^{1m}$.

January 15th.—Eating and sleeping pretty well. No discharges from the sores, which are rapidly cleaning and filling. Mind perfectly clear; memory reaches farther back. Urine voluntary and natural. Saturday night mother was frightened by a peculiar lifeless expression of face; she complained of being so tired; asked to be turned over; dropped to sleep; in early morning called for bed-pan, and with aid of enema passed good stool, and was better all day Sunday. Asked for onions and vinegar. Can now move her feet, first time in four weeks. Epistaxis this morning; slight offensive odor (menses should appear to-day). Complains of heat, but chills if uncovered; easy tears. Yellow leucorrhœa discharge to-day. Puls.$^{1m}$, one dose, dry.

January 17th.—Since last visit and the Puls. has slept longer at a time; mentally clear; remembers my last visit but forgets recent events, viz.: asks questions over again which have been answered. Openings all around look healthy and are smaller, and only a slight oozing of lymph from the right trochanter opening, which is about one and one-half inches in diameter and circular. Skin edges clean. The one opening left (on left flank) slight discharge of creamy pus. Coccygeal opening one-half inch beginning to scab. Openings over left border of sacrum but little changed in size, are also inclined to scab, but this gets rubbed off. Normal micturition but blackish urine; stool each morning by aid of enema; cannot straighten limbs; thinks the feet are fastened to the bed, but can move them slightly. A white appearance of membrane on mucous surface of vulvar labia. Breath from nostrils offensive and bloody; offensive masses at times are discharged; tongue, a white coat, but moist.

Remembering the early abuse of Carbolic-acid in this patient, I gave Carbolic-ac.$^{1m}$, three doses in water.

January 23d.—Has been improving in every way since the 17th. The odor left nostrils; the white tongue cleaned off; the urine became normal in color and quantity. The bowels have moved normally the last two days; chalky in color. Headache came on last evening at eight o'clock, forehead and temples; very tired; often waked from sleep complaining of hands and feet being numb; on left side sores look clean and healthy but not closing up (seemingly in *statu quo*); they are more sensitive to touch. ℞. Lachesis $^{1m}$, one dose.

January 24th, eve.—Saw patient; temperature and pulse normal, as also action of kidneys and bowels. All openings in skin slowly growing smaller or healing by scabbing. Through these openings, viz., at crest of left ilium, posterior to left trochanter, and all around the right trochanter, the muscles plainly visible, their action beautifully shown through the openings by sliding the skin above over the tissues underneath, contractions voluntary and involuntary are seen. The circular opening over coccyx showed the decussating fascicles of the muscles, and that one over, or rather around the right trochanter, owing to the emaciation, allows the trochanter to project through; you see that since the Lachesis the soreness is gone, and even rough handling allowed. The nose (I could now examine for the first time with consent of patient) shows septum ulcerated and turbinated bones partially necrosed. ℞. No medicine.

February 3d.—Report is good in general. The septum narium came away; nostrils communicate as far as we can see. Openings in skin gradually smaller; appetite fair; can move the limbs more, but don't like to move or try to get up. Says feet feel like lead; shows improvement in amount of flesh. Psorinum$^{50m}$.

February 7th.—Reports no particular change, condition about the same. Sulph.$^{cm}$, four doses, one every two hours.

February 22d.—After the Sulph. on February 7th, began to feel poorly, like chills, every day about noon till one o'clock, followed by fever; sweat in the night during chill; restless; no better from heat; during the heat was thirsty and drowsy; sweat during sleep. These chills, etc., lasted three days, followed by good progress in all particulars, increase in flesh. From the sore spot over the tip of coccyx a projection of tissue "like the tip of finger," not sensitive to touch. Opening over right trochanter (about one inch in diameter) for a few days has discharged yellow, watery, fetid pus, and red around edges; bowels moved naturally until last three days, constipated. Pyrogen.$^{cm}$, one dose, and if not better in four days to take Silicea$^{cm}$, one dose.

March 5th.—The color and odor of discharges improved, and she did not get the Silicea; left trochanter opening "weeping" bloody, watery, and sore. Is nervous, fidgety, and restless on damp days. Appetite changeable, but strength is better, although she don't like to sit up because of pain. No sign of menses, due the 15th. ℞. I order the Silicea to be given, and if no better, Asaf.$^{1m}$.

March 14th.—After the Silicea on the 6th, mentally more cheerful, discharges ceased, bowels moved regularly; good sleep nights until last Saturday was restless; the openings discharged yellow pus. The mother prepared the No. 2 (Asafet.$^{1m}$) and gave three doses; did not give the fourth because of increase of symptoms on that day. These subsided on Sunday. Bowels now move every other day; craves apples; no menses; cannot support weight on legs; sometimes when on back involuntary urine, stopped at once if she turns on her face or side; there is some smarting from the urine. "Has had leucorrhœa a year, none now." I gave Medorrhinum$^{cm}$, four powders, one each night.

March 22d.—Better in all particulars.

April 4th.—Gradual steady improvement. No menstrual signs; breath foul; tongue shows imprint of teeth; dirty white greenish-pale spots on dressing from sores. ℞. Merc-viv.$^{1m}$, one dose, dry.

April 14th.—Progressing in strength, flesh, and condition generally. No discharges from sores. No menses yet. No medicine.

April 24th.—Tendons feel too short; cannot yet make legs straight, but an improvement. No medicine.

May 1st.—Has been up on crutches, and has been out on tricycle, which she can propel herself.

