# Correction

**Published:** 1895-11

**Authors:** 


Correction: In the October number, at page 462, a very serious error occurs. All that portion of the page, beginning with paragraph dated May 9th and ending with the one dated June 16th, both included, should be placed at the end of the article on page 467.

